# Aetiology of Type 2 diabetes in people with a ‘normal’ body mass index: testing the personal fat threshold hypothesis

**DOI:** 10.1042/CS20230586

**Published:** 2023-08-31

**Authors:** Roy Taylor, Alison C. Barnes, Kieren G. Hollingsworth, Keaton M. Irvine, Alexandra S. Solovyova, Lucy Clark, Tara Kelly, Carmen Martin-Ruiz, Davide Romeres, Albert Koulman, Claire M. Meek, Benjamin Jenkins, Claudio Cobelli, Rury R. Holman

**Affiliations:** 1 Magnetic Resonance Centre, Translational and Clinical Research Institute, Faculty of Medical Sciences, Newcastle University, Newcastle upon Tyne, U.K.; 2 Faculty of Medical Sciences Professional Services, Newcastle University, U.K.; 3 BioScreening Core Facility, Campus for Ageing and Vitality, Faculty of Medical Sciences, Newcastle University, U.K.; 4 Department of Endocrinology, University of Virginia, Charlottesville, VA, U.S.A.; 5 Wellcome Trust-MRC Institute of Metabolic Science, University of Cambridge, Box 289, Cambridge Biomedical Campus, Cambridge, U.K.; 6 Wolfson Diabetes and Endocrine Centre, Cambridge Universities NHS Foundation Trust, Cambridge, U.K.; 7 Department of Woman and Child's Health, University of Padova, Italy; 8 Diabetes Trials Unit, Radcliffe Department of Medicine, University of Oxford, Oxford, U.K.

**Keywords:** de novo lipogenesis, hepatic steatosis, remission, triglyceride, type 2 diabetes, weight loss

## Abstract

Weight loss in overweight or obese individuals with Type 2 diabetes (T2D) can normalize hepatic fat metabolism, decrease fatty acid oversupply to β cells and restore normoglycaemia. One in six people has BMI <27 kg/m^2^ at diagnosis, and their T2D is assumed to have different aetiology. The Personal Fat Threshold hypothesis postulated differing individual thresholds for lipid overspill and adverse effects on β-cell function. To test this hypothesis, people with Type 2 diabetes and body mass index <27kg/m^2^ (*n* = 20) underwent repeated 5% weight loss cycles. Metabolic assessments were carried out at stable weight after each cycle and after 12 months. To determine how closely metabolic features returned to normal, 20 matched normoglycemic controls were studied once. Between baseline and 12 months: BMI fell (mean ± SD), 24.8 ± 0.4 to 22.5 ± 0.4 kg/m^2^ (*P*<0.0001) (controls: 21.5 ± 0.5); total body fat, 32.1 ± 1.5 to 27.6 ± 1.8% (*P*<0.0001) (24.6 ± 1.5). Liver fat content and fat export fell to normal as did fasting plasma insulin. Post-meal insulin secretion increased but remained subnormal. Sustained diabetes remission (HbA_1c_ < 48 mmol/mol off all glucose-lowering agents) was achieved by 70% (14/20) by initial weight loss of 6.5 (5.5–10.2)%. Correction of concealed excess intra-hepatic fat reduced hepatic fat export, with recovery of β-cell function, glycaemic improvement in all and return to a non-diabetic metabolic state in the majority of this group with BMI <27 kg/m^2^ as previously demonstrated for overweight or obese groups. The data confirm the Personal Fat Threshold hypothesis: aetiology of Type 2 diabetes does not depend on BMI. This pathophysiological insight has major implications for management.

## Introduction

Type 2 diabetes affects at least 10.5% of the world population yet the underlying cause of this common condition remains unresolved [1]. The disease is regarded as being heterogenous, with causes involving obesity, genetics, the microbiome, inflammation, ageing and other factors. Aetiology in people with a normal body mass index (BMI) is believed to be different from that in obese people [2], and guidelines do not recommend weight loss for management [3]. Observation that people of any BMI between 27 and 45 kg/m^2^ could return to normal metabolic control by losing a similar amount of weight [4,5] led to the Personal Fat Threshold hypothesis [6]. It postulated that the storage capacity of subcutaneous fat may vary markedly between individuals and that T2D occurs when an individual of any BMI can no longer store triglyceride safely, thus promoting excess liver fat accumulation, excess hepatic fat export and exposure of β cells to excess lipid [6].

The Personal Fat Threshold hypothesis postulates that people with T2D will exhibit the same pathophysiological mechanisms irrespective of BMI and may return to non-diabetic glucose control after weight loss. This is of profound importance as it would indicate that this common disease does not have heterogenous causative mechanisms dependent upon body weight. The question is also of major therapeutic importance as 1 in 6 of all newly-presenting T2D in the US and UK have a BMI <27 kg/m^2^ [7] (personal communication Prof Sarah Wild).

The Reversal of Type 2 diabetes Upon Normalization of Energy intake in non-obese people (ReTUNE) Study was designed explicitly to test the Personal Fat Threshold hypothesis. The prior prediction of this hypothesis was that people with normal or near-normal BMI could achieve remission of T2D by weight loss and that this would be achieved with the same underlying pathophysiological changes [6]. The effect of stepwise weight loss in people with T2D and BMI 21–27 kg/m^2^ was, therefore, examined to determine possible thresholds for correction of the underlying mechanisms, using application of MRI in conjunction with detailed pathophysiologic studies. Additionally, durability of remission was examined by re-study at 12 months.

## Methods

### Participant details

Participants with T2D of <6 years duration aged 20–70 years and not on insulin therapy were recruited between 31.5.18 and 2.11.21 with follow-up to 25.1.22. Clinical characteristics are listed in Table 1. To determine how closely to normal the metabolic features of the T2D cohort became, normoglycaemic control participants with no first-degree family history of T2D were studied on one occasion only. Hence, BMI of the controls was matched to the post-weight loss BMI of the T2D cohort and they were also matched for sex and age. Both T2D and control groups were recruited by newspaper advertisement.

**Table 1:** Change in anthropometry after each cycle of weight loss in the T2D group

	Baseline, *n*=20	8 weeks, *n*=20	16 weeks, *n*=18	24 weeks, *n*=9	52 weeks, *n*=15	Control, *n*=20
Weight (kg)	71.8 ± 2.8	67.0 ± 2.7 *P*=0.0001	65.3 ± 2.9 *P*=0.0001	62.6 ± 4.2 *P*=0.0001	64.1 ± 2.8 *P*=0.0001	61.5 ± 2.5 *P*=0.496
BMI (kg/m^2^)	24.8 ± 0.4	23.2 ± 0.4 *P*=0.0001	22.4 ± 0.4 *P*=0.0001	21.3 ± 0.5 *P*=0.0001	22.4 ± 0.5 *P*=0.0001	21.5 ± 0.5 *P*=0.226
Change in BMI (%)	-	−6.6-± 0.5	−9.9-± 0.5	−14.1 ± 1.0	−9.9 ± 1.1	–
Fat free mass (kg)	49.0 ± 2.6	48.0 ± 2.6 *P*=0.004	47.5 ± 2.8 *P*=0.0001	46.6 ± 3.8 *P*=0.0001	47.1 ± 3.0 *P*=0.0001	46.4 ± 2.2 *P*=0.845
Total body fat (%)	32.1 ± 1.5	28.8 ± 1.6 *P*=0.0001	27.7 ± 1.5 *P*=0.0001	25.8 ± 2.3 *P*=0.0001	27.7 ± 1.8 *P*=0.0001	24.6 ± 1.5 *P*=0.214
Female	36.3 ± 0.9	33.2 ± 1.0 *P*=0.0001	31.4 ± 1.0 *P*=0.0001	29.5 ± 1.7 *P*=0.0001	32.0 ± 0.9 *P*=0.0001	27.5±1.7 *P*=0.037
Male	24.2 ± 1.2	20.6 ± 1.3 *P*=0.0001	20.3 ± 1.5 *P*=0.0001	–	19.2 ± 1.8 *P*=0.006	19.2 ± 1.7 *P*=0.983
Waist circumference (cm)	90.0 ± 2.1	83.4 ± 1.9 *P*=0.001	80.9 ± 2.1 *P*=0.001	77.2 ± 2.7 *P*=0.001	79.7 ± 2.0 *P*=0.001	75.6 ± 1.8 *P*=0.140
Female	85.6 ± 2.0	79.7 ± 1.6 *P*=0.001	76.8 ± 1.7 *P*=0.001	73.6 ± 2.0	76.4 ± 1.4 *P*=0.001	71.1 ± 1.5 *P*=0.022
Male	98.1 ± 3.0	90.3 ± 3.2 *P*=0.001	89.0 ± 3.5 *P*=0.001	–	86.8 ± 3.8 *P*=0.001	83.9 ± 2.1 *P*=0.534
VAT (cm^2^)	143.2 ± 17.7	104.9 ± 14.2 *P*=0.001	95.0 ± 15.1 *P*=0.001	78.7 ± 25.3 *P*=0.001	89.7 ± 14.4 *P*=0.001	51.2 ± 13.1 *P*=0.057
Female	107 ± 12	79 ± 10 *P*=0.0001	71 ± 11 *P*=0.0001	56 ± 16 *P*=0.001	70 ± 9 *P*=0.001	26 ± 6 *P*=0.001
Male	211 ± 34	153 ± 33 *P*=0.001	136 ± 33 *P*=0.0001	–	130 ± 35 *P*=0.011	98 ± 30 *P*=0.504
SAT (cm^2^)	201.3 ± 13.5	157.6 ± 11.8 *P*=0.001	144.8 ± 12.2 *P*=0.001	125.2 ± 17.4 *P*=0.001	147.9 ± 13.4 *P*=0.001	107.4 ± 11.9 *P*=0.031
Female	216 ± 17	168 ± 13 *P*=0.002	160 ± 12 *P*=0.0001	133 ± 19 *P*=0.003	159 ± 14 *P*=0.0001	110 ± 16 *P*=0.034
Male	174 ± 18	138 ± 22 *P*=0.001	120 ± 25 *P*=0.001	–	127 ± 29 *P*=0.005	102 ± 18 *P*=0.502

Mean ± SEM. Number stated is for the whole cohort of 13 female and 7 male participants. Male data are not shown for 24 weeks (third cycle) as *n*=3. The *P*-values for each time point vs. baseline are shown. For the control data, *P* refers to comparison with 52 week time point (P4).

#### Protocol

All clinical studies were conducted at the Newcastle University Magnetic Resonance Centre. As monogenic and autoimmune diabetes are more common in this ‘normal’ weight group, testing was carried out after informed consent to ensure that other types of diabetes were excluded. Screening was carried out for monogenic diabetes (www.diabetesgenes.org) and islet cell antibodies (GAD (U/ml, positive >10.9), human islet cell IA2 (U/ml, positive >7.49) or ZnT8 (U/ml, positive >9.99)) in the UK national test centre [8]. The study design was single group analysis of change from baseline. The cohort of 20 participants with T2D of duration <6 years and not on insulin therapy underwent 2 cycles of 5% rapid weight loss. If the HbA_1c_ remained above 48 mmol/mol [9] and if the individual wished, a third cycle was undertaken. One person was in remission after the first cycle of weight loss and wished to remain at that weight. Each cycle consisted of 2–4 weeks low calorie diet (600 kcal/day of formula meal replacements plus up to 200 kcal/day of non-starchy vegetables) [4] followed by 4–6 weeks weight stability with advice about types and quantities of foods, predominantly following a Mediterranean style eating pattern. Studies were conducted at baseline on usual hypoglycaemic agent therapy then at 8, 16 and 24 weeks after weight loss and weight stability. The weight loss and weight maintenance phases were supervised by one-to-one contact with a research dietitian (A.C.B. or K.M.I.). To determine durability of change of metabolic state, studies were also conducted at 52 weeks with dietetic review at 9 months, with telephone advice if required.

The 10-year risk of heart attack or stroke at baseline and 52 weeks in the T2D cohort and in the controls was calculated using QRISK3 [10]. Diary-based total dietary intake over 3-day periods were evaluated using Intake 24 [11] during the 2 weeks prior to each study visit (baseline, 8, 16, 24 and 52 weeks). Average intake of macronutrients, fruit and vegetables during the study are shown in Supplementary Table S3.

#### Determination of liver fat content

Liver fat content was quantified by the three-point Dixon magnetic resonance (MR) method, with gradient-echo scans acquired during breath holding using a 3T Philips Achieva scanner with a sixteen-channel torso array (Philips, Netherlands) [12]. Homogenous regions of interest were selected on five image slices of liver.

#### Determination of visceral and subcutaneous fat content

Three-point Dixon MRI was also acquired at the level of the L2-L3 vertebral space to estimate subcutaneous and visceral fat (SAT, VAT). Thresholding and watershed analysis using ImageJ were applied to calculate VAT and SAT areas in cm^2^ at L2-L3 from the proton density fat fraction map.

#### Determination of liver-derived lipoprotein triglyceride

A Sorvall MX150+ microcentrifuge (Thermo Scientific) with S140-AT rotor was used for lipoprotein separation. Chylomicrons were separated from plasma using a supernate solution of 1.006 g/ml at 12,000 rpm for 30 min. The VLDL-TG fraction was then separated at 140,000 rpm for 50 min with supernate solution of 1.006 g/ml. Triglyceride concentrations in the fractions were quantified using the standard method (Roche Diagnostics, U.K).

#### Determination of indices of de novo lipogeneis

Concentration of plasma triglyceride 48:1 and 50:1 were used as best estimates of rate of fasting *de novo* lipogenesis [13] measured by liquid chromatography hyphenated with high-resolution mass spectrometry detection (LC-MS) [14].

#### Calculation of insulin sensitivity and insulin secretion

Indices of insulin sensitivity (SI) and insulin secretion normalised to insulin sensitivity (DI) were estimated by mathematical modelling [15] after a standard meal of ordinary foodstuffs (587 kcal; 65% carbohydrate, 13% protein and 19% fat) following an overnight fast. Total cholesterol, HDL cholesterol and blood pressure were measured. HOMA2_IR was estimated using the Oxford Diabetes Trials Unit HOMA Calculator [16].

#### Plasma adipokine assay

Plasma adipokine levels were assessed using R&D sandwich ELISA kits (Bio-Techne, Abingdon, U.K.), in quadruplicate on a 384-well format. All colorimetric reactions were read at 450 nm with correction at 540 nm on a FLUOstar Omega spectrofluorometer (BMG–LABTECH) and evaluated by the corresponding software MARS Data Analysis Version 1.20. Four parameter logistic (4-PL) curve-fits were applied for all standard curves; concentration values were corrected against an internal control as well as the corresponding dilution factor for each biomarker.

Glucose was measured by the oxidase method (Yellow Springs Inc., U.S.A.). HbA_1c_ was quantified using HPLC (Tosoh Bioscience, U.K.). C-peptide, insulin, glucose, NEFA, VLDL triglyceride and other metabolites were analysed at a Clinical Pathology Accredited Laboratory (Newcastle upon Tyne Hospital NHS Foundation Trust, U.K.).

### Statistical analysis

Power calculation: The study was powered on primary dual outcome measures of reversal to non-diabetic HbA_1c_ and change in HbA_1c_ from baseline to post-diet because the Personal Fat Threshold hypothesis depends upon improvement in glucose control. In the absence of prior information on a non-obese group, a clinically relevant reversal rate was taken as 50%. Twenty-one participants (not including dropouts or exclusion due to other specific diagnosis) were allowed to calculate a confidence interval of width 0.4, assuming an actual reversal rate of 50%, i.e. 50% (95% CI: 30% to 70 [DS1]%). Twenty four participants were recruited to allow for dropouts or exclusions. A type 1 error of 5% and power of 90% was used. The co-primary outcome was the change in HbA_1c_ from entry/pre-weight loss to post-weight loss. As no data existed on a non-obese group, data from the short duration group of the Counterbalance study (mean BMI: 34 kg/m^2^) were used (a clinically relevant mean change in HbA_1c_ pre- and post-weight loss of 0.6% with SD = 0.8%) [26].

Analyses were conducted on all subjects with paired data before and after weight loss. For physiological parameters, the principal outcomes were HbA_1c_ change from baseline to <48 mmol/mol at CEP and at 12 months. Data are presented as mean ± SEM, mean ± SD or median (IQ range), if not normally distributed. Student paired or two-sample *t-*tests were used as appropriate for parametric data and the Wilcoxon-Rank test or Mann–Whitney *U-*test for paired or unpaired nonparametric data. Analyses were performed using Minitab 17 (Minitab, U.S.A.) and Stata, version 13.1 (StataCorp LP, College Station, Texas, U.S.A.). For the time series data, Benjamini–Hochberg testing was carried out using a false discovery rate of 0.1 to detect multiple testing errors as indicated in the tables. Three participants were recruited late due to COVID-19 and were studied only to the 24-week time point due to grant period constraint. Missing data are indicated in each of the figures and tables. A two-tailed *P-*value <0.05 was considered as significant.

## Results

### Clinical and metabolic characteristics

Of 24 individuals screened, four were found to have non-T2D aetiology and excluded, two with glucokinase-deficient monogenic diabetes and two with Type 1 diabetes. At baseline, there were 20 individuals in the T2D cohort (13 females and 7 males) who were (mean ± SD) aged 59.0 ± 7.0 years, weighed 71.8 ± 12.6 kg, and had a mean BMI of 24.8 ± 1.7 (range 21.2–26.9) kg/m^2^ (Table 1). Of these, 16 were White European, three were South Asian and one was of Middle Eastern ethnicity. Mean diabetes duration was 2.8±1.9 years. Eleven had a first-degree family history of T2D and 12 were taking oral glucose-lowering agents (metformin 10, gliclazide 2). Twenty individuals in the control group (13 females and 7 males) were matched for weight following weight loss in the T2D group. They were aged 58.0 ± 10.5 years, weighed 61.5 ± 11.3 kg, and had a mean BMI of 21.5 ± 2.2 kg/m^2^ (Table 1). There were 17 White European, 2 of South Asian and 1 of Far Eastern ethnicity.

### Weight loss

**Figure 1:**
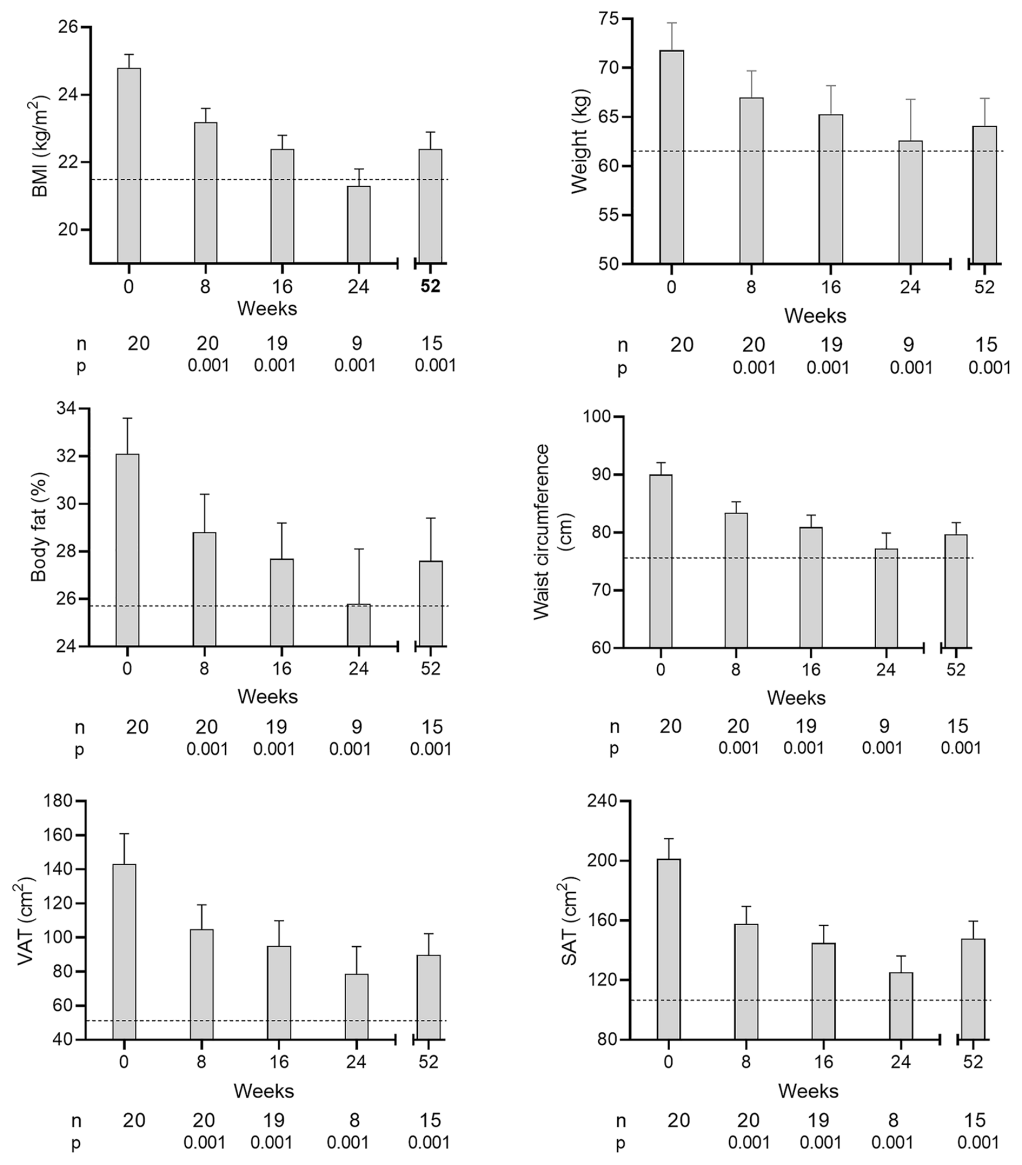
Time course of change in major clinical parameters after each cycle of weight loss and at 12 months Changes in mean (+SEM) BMI, weight, % body fat, waist circumference, visceral adipose tissue (VAT) and subcutaneous adipose tissue (SAT) in the T2D group during the intervention at each time point. Numbers of participants and level of statistical significance vs. baseline are shown below each time point. The dotted line shows the mean control values for each parameter.

In the T2D group mean body weight decreased as *per* protocol at each step from 71.8 ± 2.8 kg (Figure 1 and Table 1) and remained stable between the final weight loss cycle visit (cycle 2 or 3) and 12 months (63.1 ± 3.0 vs. 64.1 ± 2.8 kg, *P*=0.88). Total body fat mirrored this (32.1 ± 1.5% at baseline and 27.7 ± 1.8% at 12 months, *P*=0.0001, Figure 1 and Table 1) remaining greater than control in women (32.0 ± 0.9% vs. 27.5 ± 1.7%, *P*=0.037) but not in men (19.2 ± 1.8% vs. 19.2 ± 1.7%, *P*=0.983, Table 1).

### Physiological changes of remission

#### Liver fat

**Figure 2:**
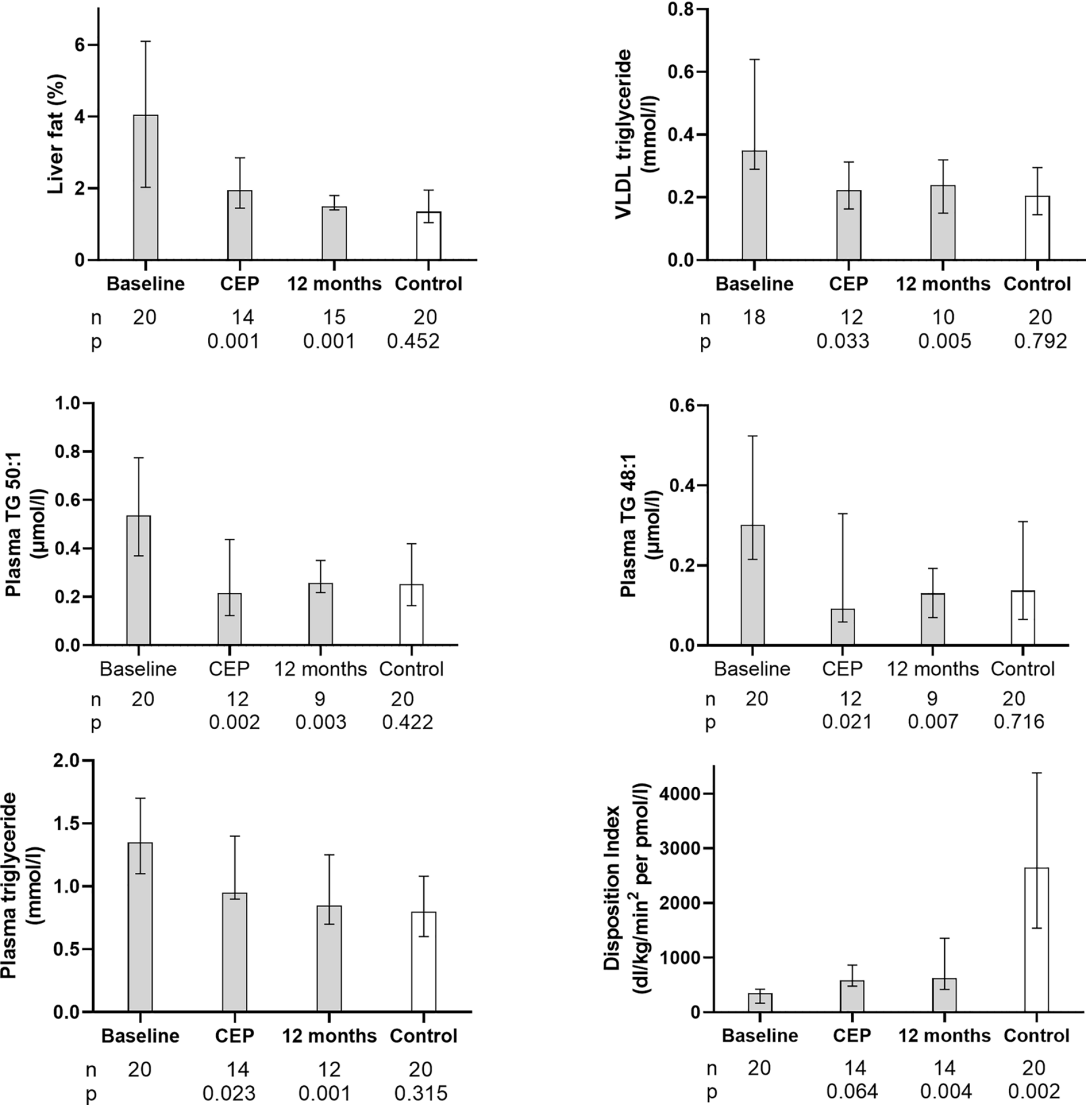
Major pathophysiological parameters at baseline, at the Clinical End Point of HbA1c <48mmol/mol (CEP) and at 12 months Median (IQR) liver fat, very low-density lipoprotein-1 (VLDL) triglyceride, plasma triglycerides with palmitic acid content reflecting rate of *de novo* lipogenesis, total plasma triglyceride, and Disposition Index at baseline, the co-primary end points of CEP (clinical end point; HbA_1c_ <48 mmol/mol) and at 12 months in the T2D group. Numbers of participants and level of statistical significance vs. baseline are shown below CEP and 12 months. Non-diabetic control data are also shown with the level of significance vs. T2D at 12 months below ‘Control’.

Median (interquartile range) liver fat content decreased from 4.0 (2.1–6.0)% to 1.8 (1.5–2.8)% at the clinical endpoint (CEP: achievement of HbA_1c_ <48 mmol/mol) (*n*=14, *P*<0.001, Figure 2). It decreased further with increasing weight loss and at 12 months was 1.5 (1.4–1.8)% (*P*=0.001 vs. baseline), comparable to control group levels (1.3 [1.0–1.8)%, *P*=0.452).

#### De novo lipogenesis

Median plasma 50:1 triglyceride decreased from baseline of 0.54 (0.37–0.77) to 0.22 (0.12–0.44) µmol/l (*P*=0.002) at CEP. It remained low at 12 months (0.26 [0.22–0.35] µmol/l (*P*=0.003 vs. baseline), and similar to control levels (0.25 [0.16–0.42], *P*=0.496, Figure 2). Plasma 48:1 triglyceride decreased from baseline of 0.30 (0.22–0.53) to 0.09 (0.06–0.33) µmol/l (*P*=0.021) at CEP. It was 0.13 (0.07–0.19) µmol/l at 12 months (*P*=0.007 vs. baseline) when it did not differ from control levels 0.14 (0.07–0.31) µmol/l (*P*=0.426, Figure 2). The time course of change for both triglycerides is shown in Supplementary Figure S1.

#### VLDL and plasma total triglyceride

Median fasting plasma VLDL triglyceride was 0.35 (0.30–0.61) mmol/l at baseline and 0.22 (0.17–0.30) mmol/l at CEP (*P*<0.033), similar to the control group (0.21 [0.15–0.29] mmol/l,* P*=0.555, Figure 2 and Table 3). At 12 months, it was 0.24 (0.17–0.32) mmol/l (*P*<0.005 vs. baseline; *P*=0.792 vs. controls). Median fasting plasma total triglyceride decreased in parallel from 1.4 (1.1–1.7) to 1.0 (0.9–1.3) mmol/l at CEP (*P*=0.023) and to 0.9 (0.7–1.1) mmol/l at 12 months (*P*=0.001), when it was similar to the control group (0.8 [0.6–1.1] mmol/l, *P*=0.315).

#### β-Cell function

Median fasting plasma insulin decreased from 36 (26–53) pmol/l to 25 (18–36) pmol/l at CEP (*P*=0.001). It was 22 (15–31) pmol/l at 12 months (*P*=0.001), similar to the control group (20 [11–32] pmol/l, *P*=0.716). Fasting C-peptide decreased from 0.58 (0.53–0.99) nmol/l at baseline to 0.48 (0.43–0.70) nmol/l at CEP (*P*=0.001) and to 0.45 (0.35–0.58) at 12 months (*P*=0.006), similar to the control group (0.45 [0.36–0.55] nmol/l, *P*=0.532). Meal-related insulin secretion corrected for insulin sensitivity (disposition index, DI) was 348 (166–423) dl/kg/min^2^ per pmol/l at baseline and 583 (410–803) dl/kg/min^2^ per pmol/l at CEP (*P*=0.064), increasing to 624 (437–1342) dl/kg/min^2^ per pmol/l at 12 months (*P*=0.006 vs. baseline). At 12 months, it was within the range of the control group values (586–8728 dl/kg/min/pmol/l) but remained significantly lower than the median control group value (2652 [1546–4190] dl/kg/min/pmol/l,* P* <0.001) (Figure 2). Supplementary Figure S1 shows the time course of change in major pathophysiological factors.

#### Insulin sensitivity and postprandial metabolism

Fasting insulin resistance, assessed as HOMA2_IR, reflecting insulin control of hepatic glucose output, decreased from 2.02 (1.44–3.30) to 1.32 (0.97–1.97) at CEP (*P*=0.001) and to 0.88 (0.51–1.36) at 12 months (*P*=0.001) when it did not differ significantly from the control group (0.67 [0.47–1.10], *P*=0.342). Mean meal related insulin sensitivity, modelled as SI, was 9.9 ± 1.4 at baseline and 16.6 ± 3.85 × 10^−5^ dl/kg/min per pmol/l at CEP (*P*=0.088), increasing at 12 months to 19.2 ± 4.0 × 10^−5^ dl/kg/min per pmol/l (*P*=0.022) but still significantly lower than the control group (36.3 ± 5.3 × 10^−5^ dl/kg/min per pmol/l, *P*=0.015). Median AUC_glucose_ decreased from 2162 (2024–2498) at baseline to 1644 (1486–2011) mmol/l min at CEP (*P*=0.016) and 1795 (1559–1924) mmol/l min at 12 months (*P*=0.011), remaining higher than the control group (1153 [1038–1232] mmol/l min, *P*<0.001). Mean fasting plasma NEFA at baseline and 12 months did not differ significantly (0.72 ± 0.04 vs. 0.57 ± 0.04mmol/l, *P*=0.155) and suppressed to a similar extent following the standard test meal (maximum suppression 0.13 ± 0.02 vs. 0.10 ± 0.01mmol/l, *P*=0.446) (Supplementary Table S1). Mean fasting plasma ketones (measured after each period of weight stability) did not change significantly (0.19 ± 0.02 at baseline vs. 0.30 ± 0.07 mmol/l at 12 months, *P*=0.150) and were not different from the control group (0.24 ± 0.03mmol/l, *P*=0.361, Table 2).

**Table 2:** Change in fasting plasma lipids

	Baseline	8 weeks	16 weeks	24 weeks	52 weeks	Controls
Total triglyceride (mmol/l)	1.4 (1.1–1.7)	1.2 (0.9–1.5) *P*=0.0110	0.9 (0.8–1.2) *P* < 0.0001	0.9 (0.6–1.1) *P*<0.0001	0.9 (0.7–1.1) *P*=0.0042	0.8 (0.6–1.1) *P*=0.520
VLDL triglyceride (mmol/l)	0.35 (0.30–0.61)	0.30 (0.19–0.50) *P*=0.186	0.22 (0.15–0.29) *P*=0.001	0.22 (0.16–0.24) *P*=0.002	0.24 (0.17–0.32) *P*=0.021	0.21 (0.15–0.29) *P*=0.676
Chylomicron triglyceride (mmol/l)	0.33 ± 0.4	0.28 ± 0.05 *P*=0.012	0.20 ± 0.03 *P*=0.0001	0.16 ± 0.04 *P*=0.002	0.27 ± 0.08 *P*=0.128	0.23 ± 0.03 *P*=0.597
Total cholesterol (mmol/l)	4.7 ± 0.2	4.4 ± 0.2 *P*=0.042	4.1 ± 0.2 *P*=0.003	4.3 ± 0.3 *P*=0.073	4.4 ± 0.2 *P*=0.103	4.9 ± 0.3 *P*=0.115
HDL cholesterol (mmol/l)	1.4 ± 0.1	1.4 ± 0.1 *P*=1.000	1.4 ± 0.1 *P*=0.163	1.7 ± 0.1 *P*=0.004	1.6 ± 0.1 *P*=0.001	1.7 ± 0.1 *P*=0.255
LDL cholesterol (mmol/l)	2.9 ± 0.2	2.5 ± 0.2 *P*=0.081	2.2 ± 0.2 *P*=0.007	2.3 ± 0.2 *P*=0.280	2.4 ± 0.2 *P*=0.207	2.8 ± 0.3 *P*=0.244
Cholesterol/HDL ratio	3.4 ± 0.2	3.3 ± 0.1 *P*=0.185	2.9 ± 0.1 *P*=0.001	2.6 ± 0.2 *P*=0.024	2.8 ± 0.2 *P*=0.001	2.9 ± 0.2 *P*=0.595
Non-esterified fatty acids (mmol/l)	0.72 ± 0.04	0.60 ± 0.05 *P*=0.077	0.61 ± 0.04 *P*=0.185	0.82 ± 0.13 *P*=0.732	0.57 ± 0.04 *P*=0.155	0.6 ± 0.1 *P*=0.463
Ketones (mmol/l)	0.19 ± 0.02	0.24 ± 0.02 *P*=0.049	0.38 ± 0.11 *P*=0.087	0.40 ± 0.07 *P*=0.021	0.30 ± 0.07 *P*=0.150	0.23 ± 0.02 *P*=0.361

Mean ± SEM or median (IQR). Significance stated as paired analysis from baseline for each time point (all confirmed by Benjamini–Hochberg correction). The *P-*values for each time point versus baseline are shown. Significance of difference shown in control column is in comparison with the 52-week T2D time point.

#### Plasma adipokines

Median PAI-1 decreased from 8.64 (6.41–11.22) ng/ml at baseline to 6.32 (4.68–7.96) ng/ml at CEP (*P*=0.009, compared with controls 4.87 [3.49–8.47] ng/ml, *P*=0.230). At 12 months (6.31 [4.63–9.36] ng/ml) it did not differ from control (*P*=0.250). Median leptin decreased from 442 (266–744) to 296 (75–447) ng/ml at CEP (*P*=0.002) when it did not differ from the control group (227 (162–290) ng/ml, *P*=0.517). It remained similar at 12 months (299 [123–513) ng/ml, *P*=0.001 vs. baseline and* P* =0.474 vs. controls). Median IL-6, FGF-21, GDF-15 and hsCRP values did not change significantly to CEP. IL-6 and hsCRP decreased significantly and adiponectin increased significantly at 12 months only (Table 3).

**Table 3:** Adipokines at baseline and after each weight loss period

	Baseline	8 weeks	16 weeks	24 weeks	52 weeks	Controls
	*n*=20	*n*=20	*n*=19	*n*=8	*n*=15	*n*=20
PAI-1 (ng/ml)	8.64 (6.41–11.22)	5.99* (3.67–7.84) *P*=0.006	5.44 (3.65–8.69)* *P*<0.001	4.94 (4.23–8.52)* P=0.008	6.31 (4.63–9.36)* *P*=0.007	4.87 (3.49–8.47) *P*=0.250
hsCRP (ng/ml)	815 (556–1951)	854 (475–1302) *P*=0.048	848 (406–2595) *P*=0.829	654 (246–765) *P*=0.195	386 (281–751) *P*=0.035	563 (225–1247) *P*=0.677
IL6 (pg/ml)	1.16 (0.88–1.72)	1.44 (0.97–1.60) *P*=0.956	1.25 (0.88–1.81) *P*=0.196	0.79 (0.66–1.94) *P*=0.039	1.06 (0.67–1.37) *P*=0.041	0.88 (0.72–1.74) *P*=0.641
Leptin (ng/ml)	442 (266–744)	312 (112–499)* *P*<0.001	224 (133–343)* P<0.001	208 (67–338)* P=0.008	299 (123–513)* *P*<0.001	227 (162–290) *P*=0.474
Adiponectin (ng/ml)	1364 (1106–2396)	1875 (1261–2587) *P*=0.202	1842 (1345–3090) *P*=0.049	2881 (1378–4518)* *P*=0.008	1854 (1596–4512)* P=0.005	2287 (1630–2727) *P*=0.934
FGF-21 (pg/ml)	425 (258–666)	308 (197–814) *P*=0.546	298 (160–619)* P=0.003	248 (130–547)* *P*=0.008	330 (114–1192) *P*=0.252	362 (249–2842) *P*=0.264
GDF-15 (pg/ml)	591 (411–879)	444 (347–555) *P*=0.105	457 (356–627) *P*=0.080	355 (341–610) *P*=0.039**†**	502 (381–736) *P*=0.978	446 (372–620) *P*=0.7260

Median (IQ range). Benjamini–Hochberg correction confirmed the significance of difference from baseline with one exception.**^†^** The *P-*values for each time point versus baseline are shown. The *P-*values for difference between Controls and the 12-month (P4) data for the type 2 diabetes participants are shown in the Controls column.

#### Glucose control

**Figure 3:**
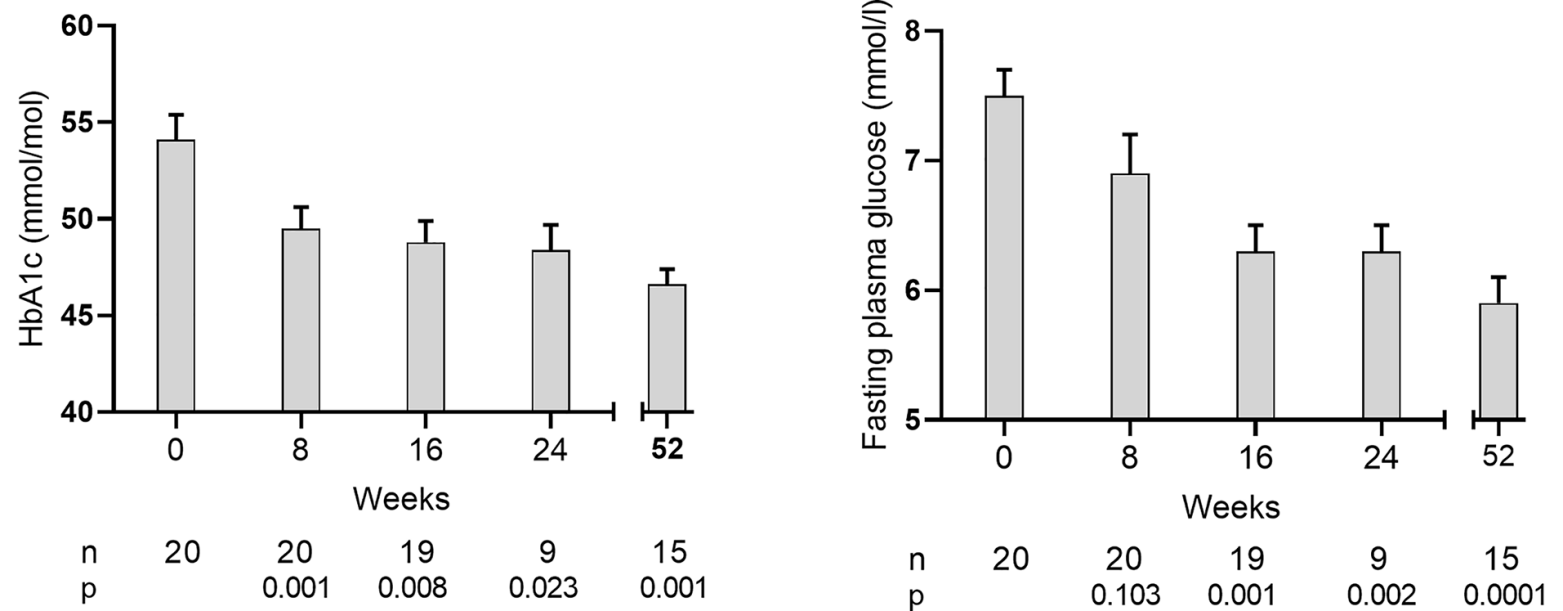
Time course of change in glycaemic measures after each cycle of weight loss and at 12 months Change in mean (+SEM) HbA_1c_ and fasting plasma glucose values in the T2D group during weight stability following each weight loss cycle and also after longer term follow up at 12 months. Numbers of participants and significance level versus baseline are shown below each time point.

HbA_1c_ decreased to <48 mmol/mol at CEP in 70% (14/20) of participants, declining from baseline of 54 ± 1mmol/mol to 46 ± 1 (*P*<0.0001) at 12 months when BMI was 22.4 ± 0.5 kg/m^2^ (Figure 3). The mean change from baseline was −8.4 ± 1.6 mmol/mol at CEP and −7.4 ± 1.7 mmol/mol at 12 months. Oral glucose-lowering agents remained discontinued throughout the study in those achieving HbA_1c_ <48 mmol/mol and were restarted in one participant remaining >48 mmol/mol. The threshold of remission off all glucose-lowering agents was achieved with median weight loss of 6.5 (range: 5.5–10.2)%.

Mean fasting plasma glucose decreased from baseline of 7.4 ± 0.2 to 5.9 ± 0.1mmol/l (*P*=0.001) at CEP and to 5.8 ± 0.2 mmol/l at 12 months (*P*=0.0001 vs. baseline) (Figure 3). The CEP was achieved following median (IQR) weight loss of 4.7 (3.4–9.2) kg, equating to 6.4 (5.4–11.1)% below baseline. The median time to first HbA_1c_ value <48 mmol/mol was 8 (8–10) weeks (*n*=10 at 8 weeks, 3 at 16 weeks, and 1 at 52 weeks).

There were no significant baseline differences in HbA_1c_ between those achieving or not achieving the CEP, although all 6 who did not achieve CEP were taking an oral glucose-lowering agent at baseline compared with 6/14 of those who achieved CEP (Table 2). In particular, there were no significant differences in BMI, sex, age, ethnicity or HOMA2_IR (Supplementary Table S2). Changes in composition of food intake during the study is shown in Supplementary Table S3.

### Lipid-related and vascular factors

The ratio of total cholesterol/HDL cholesterol decreased from 3.4 ± 0.2 to 3.1 ± 0.1 at CEP (*P*=0.021) and became similar to the control group at 12 months (2.8 ± 0.2 vs. 2.9 ± 0.2, *P*=0.758). Median systolic blood pressure was 131 (123–136) mmHg at baseline, 122 (115–136) mmHg at CEP (*P*=0.052 vs. baseline) and 118 (112–125) mmHg at 12 months (*P*=0.008 vs. baseline). Median diastolic blood pressure was 76 (64–81) mmHg at baseline, 72 (66–78) mmHg at CEP (*P*=0.052) and 64 (59–67) mmHg at 12 months (*P*=0.003). Antihypertensive agents were withdrawn after baseline tests were performed in the 4 participants receiving these agents but were required to be re-commenced in three participants. The 10-year cardiovascular disease risk (QRISK) decreased from 14.4 ± 1.5% at baseline to 8.1 ± 1.1% (*P*<0.001) at 12 months, when it was similar to that of the control group (6.2 ± 1.2%, *P*=0.242).

Waist circumference decreased between baseline and 12 months from 90.0 ± 2.1 to 79.7 ± 2.0 cm (*P*<0.001, Table 1 and Figure 1). Visceral adipose tissue (VAT) decreased by 37.4% and subcutaneous adipose tissue (SAT) by 26.5% (*P*=0.001 for both, Table 1 and Figure 1). In women, VAT and SAT remained higher than control (70 ± 9 vs. 26 ± 6 cm^2^, *P*=0.001 and 159 ± 14 vs. 110 ± 16 cm^2^, *P*=0.034). In men after weight loss a non-significant similar trend was evident in waist circumference, VAT and SAT (Table 1). MRI fat maps illustrating the change in VAT with weight loss compared with matched control participants are shown in Supplementary Figure S2.

## Discussion

The ReTUNE study confirms the Personal Fat Hypothesis predictions by showing that 70% of people with T2D with normal or near-normal BMI return to non-diabetic HbA_1c_ despite stopping all glucose-lowering agents after weight loss, with the same underlying pathophysiological changes as already demonstrated in overweight/obese people. These data introduce a frame shift of understanding of T2D with demonstration of homogenous aetiology irrespective of BMI. The return to normal levels of liver fat after dietary weight loss in T2D, first demonstrated by Petersen and colleagues [17], emphasises the tight relationship between liver fat content, hepatic insulin sensitivity and restraint of hepatic glucose output by insulin [18–20]. The Counterpoint and subsequent studies established that this normalization of physiology in overweight/obese people presenting with T2D was accompanied by recovery of β-cell function [4,5,21,22]. The stepwise induction of weight loss in the T2D group identified that the BMI threshold for return to non-diabetic HbA_1c_ values was crossed at a median of 23.1 kg/m^2^ with a median 6.5% weight loss, less than the 9.9% weight loss required for the higher-BMI group studied in DiRECT [23].

Although liver fat content at baseline in the present study was within the usually accepted normal range derived from the Dallas Heart Study with mean BMI 30kg/m^2^ [24], it is important to consider what is normal for people with lower BMI values. The differential between baseline liver fat content in the ReTUNE T2D group was three-fold greater than that in the control group, similar to that observed between groups of heavier people with T2D and controls [21]. Consistent with this, Petersen and colleagues have observed the 95% boundary for liver fat to be less than 2% in individuals with BMI under 25 kg/m^2^ [25]. The proximal mediators of hepatic insulin resistance are the toxic lipid intermediaries (including ceramides and diacylglycerol) rather than stored triglyceride itself [26], and the former would be expected to fall more rapidly on initiation of negative energy balance. Close study of the initial time course of change in hepatic insulin resistance in the Counterpoint study using the same hypocaloric diet showed a 30% decrease in liver fat yet complete normalisation of directly measured hepatic insulin resistance within 7 days [4]. The Counterpoint study was designed to test the specific predictions of the Twin Cycle Hypothesis [27] that T2D was caused by excess fat inside liver and supplied to the β cells and that this the underlying mechanisms could be reversed to normal by weight loss, and hence the present study provides further confirmation of fat-dependent aetiology of the disease.

The >50% decrease in plasma triglycerides containing predominantly palmitic acid reflects decreased fasting *de novo* lipogenesis [13], as previously documented with weight loss in heavier people [4,5,21,28]. Palmitic acid is the most potent fatty acid in suppressing β-cell function [29]. Although functional β-cell mass has been shown to return completely to normal during 12 months of dietary weight loss-induced remission of T2D, first phase insulin secretion improves but does not normalise either in the present or previous studies [30]. Onset of T2D is likely to relate primarily to the β-cell functional insulin secretory capacity [31], confirmed in studies of remission and recurrence with weight regain in people with wide range of T2D duration [5,21,32]. But even in genetically lipid-susceptible β cells function will not be impaired if the β-cell lipid environment remains normal. The metabolic stress of chronic nutrient oversupply *in vitro* is particularly evident following exposure to palmitate, [33], the product of *de novo* lipogenesis which is markedly increased in states of muscle insulin resistance [34]. The fat overflow hypothesis and separately the concept that chronic excess fatty acid exposure could impair β-cell function are far from new [35–39]. Our present and previous studies demonstrate that these factors act in a coordinated fashion to cause T2D, and that effective weight loss across BMI range leads to a lower rate of continuous fatty acid delivery from plasma triglycerides to β cells [4,5,22,40].

The development of hyperglycaemia will add to this β-cell metabolic insult, contributing to the well-recognised longer-term deterioration in glycaemic control [41]. De-differentiation and loss of specialised function of β cells is consistent with recent studies [42–45] and re-differentiation would explain the observed recovery when endoplasmic reticulum stress is removed [42,43,45–47]. Markers of de-differentiation are expressed in β cells from human T2D pancreases [44,48]. There is no proof that de-differentiation is the sole process underlying β-cell dysfunction, but if the state of metabolic stress persists too long, irreversible loss of endocrine function results [5,49]. The response to weight loss could be used as a phenotyping tool to guide future genetic studies of two distinct characteristics: β-cell susceptibility to lipid induced dysfunction, and β-cell durability in avoiding irreversible loss of function despite chronic fat and glucose excess. The genetic basis of heterogeneity in subcutaneous fat storage capacity itself has been confirmed [50].

A secondary aim of ReTUNE was to examine whether raised plasma adipokine concentrations could identify people who were above their personal fat thresholds. Median PAI-1 and leptin were both found to be elevated in the T2D group and to decrease once HbA_1c_ became <48 mmol/mol but a larger study will be necessary to determine whether or not a combination of PAI-1 and leptin could be a reliable indicator.

The Personal Fat Threshold paper drew attention to the median BMI of the people with newly diagnosed T2D enrolled in UK Prospective Diabetes Study (UKPDS) of 27.5 kg/m^2^, with over one-third (36%) having a normal BMI (<25kg/m^2^) [6]. When recruitment for UKPDS commenced in the 1970’s, 64% of the background population had a BMI less than 25 kg/m^2^ [51] and the apparent lack of association of T2D with obesity at that time [52] is explicable by the small numbers of people with high BMI. Now that 28% of the UK adult population have a BMI greater than 30 kg/m^2^ [53], the association of obesity with T2D is so prominent that a causal relationship has become widely assumed. Hence, the nature of T2D itself has been overlooked, and concepts of heterogeneity of aetiology in different weight groups have emerged [54]. This is partly based on the assumption that non-obese people with T2D have less insulin resistance and a greater β-cell defect compared with those who are obese [55]. However, when appropriate BMI-matched controls are used, there is no difference in these parameters between non-obese and obese people [56] and the β-cell response after test meals is similar in non-obese and obese T2D groups [57].

People with a BMI <27 kg/m^2^ account for 16% of newly diagnosed T2D [7]. Demonstration of the value of weight loss in this group carries major health economic implications. The present and previous studies show that the majority of people presenting with T2D are heavier than their individual metabolism can tolerate [4,5,21,22]. However, in the lower BMI range exclusion is necessary of monogenic or autoimmune diabetes which can mimic T2D with 4/24 people (16%) found to have other specific causes of diabetes.

Limitations of the present study must be considered. The group studied was relatively small but the effect size was so large with highly statistically significant outcomes, and this detailed physiologic study was powered to test an *a priori* hypothesis. Also, the group characteristics were typical for people with T2D and BMI <27 kg/m^2^. Secondly, the ethnicity reflected that in the North East of England and was largely white European. Remission of T2D after weight loss has been documented in all ethnicities tested [58–61], although it would be expected that different ethnicities will have different personal fat thresholds. Thirdly, the possibility of achieving the necessary weight loss with diet may be doubted by many doctors, but the acceptability of the specific dietary approach has been confirmed [62] and its success in the wider population of people with T2D has led to a NHS England national programme for remission [63]. At present this is limited to those with BMI >27 kg/m^2^. Finally, follow-up beyond one year is required for this BMI group studied, although there was a notable stability of weight during the low intensity follow-up between 6 and 12 months which differed from the weight regain observed in people similarly managed with starting BMI >27 kg/m^2^ [21].

The Personal Fat Threshold hypothesis explains the apparent weight-related heterogeneity in T2D with individual differences in capacity for fat storage in metabolically safe depots. However, the pathophysiologic mechanisms underlying T2D and its reversal are identical in people with normal or raised BMI.

## Clinical perspectives

- The study tested the Personal Fat Threshold hypothesis that Type 2 diabetes in people of BMI <27 kg/m^2^ could achieve remission by dietary weight loss, accompanied by the same underlying pathogenic mechanisms as in heavier people.
- The study group were shown to have raised liver fat, *de novo* lipogenesis and hepatic fat export compared with a matched non-diabetic control group and these parameters returned to normal resulting in improved β-cell function with 70% achieving remission lasting for 12 months.
- Recognising that Type 2 diabetes is directly caused by overnutrition, potentially reversible by dietary weight loss to below a personal threshold, is vital to direct clinical management as well as future research.

## Supplementary Material

Supplementary Figures S1-S2 and Tables S1-S3

## Data Availability

All data will be made freely available on the Mendelay database accessible at https://data.mendeley.com/datasets/72bm99t7wm/draft?a=832a3c4e-964e-4b00-ab74-89ab564675a8.

## Open Access

Open access for this article was enabled by the participation of Newcastle University in an all-inclusive *Read & Publish* agreement with Portland Press and the Biochemical Society under a transformative agreement with JISC.

## Abbreviations

AUC: area under the curve
BMI: body mass index
CEP: Clinical end point
FGF-21: fibroblast growth factor-21
HDL: high-density lipoprotein
HOMA: homeostasis model assessment
hs CRP: highly sensitive C-reactive protein
IL-6: interleukin-6
MRI: magnetic resonance imaging
PAI-1: plasminogen activator inhibitor-1
SAT: subcutaneous adipose tissue
T2D: Type 2 diabetes
VAT: visceral adipose tissue
VLDL: very low-density lipoprotein
