# Supplementary material for: Aetiology of Type 2 diabetes in people with a ‘normal’ body mass index: testing the personal fat threshold hypothesis
Source: Clin Sci (Lond). 2023 Aug 31;137(16):1333–46. doi: 10.1042/CS20230586 (PMC10472166; doi:10.1042/CS20230586)
Supplement: Supplementary Figures S1-S2 and Tables S1-S3 [file CS-2023-0586_supp.pdf]

SUPPLEMENTARY MATERIAL

STROBE Statement—checklist of items that should be included in reports of observational studies

	Item No.	Recommendation	Page No. [lines]	Relevant text from manuscript
**Title and abstract**	1	(*a*) Indicate the study's design with a commonly used term in the title or the abstract	2 [50-53]	…in a cohort ….
		(*b*) Provide in the abstract an informative and balanced summary of what was done and what was found	2 [51-60]	To test this hypothesis …
**Introduction**				
Background/rationale	2	Explain the scientific background and rationale for the investigation being reported	3 [68-84]	… the underlying…..
Objectives	3	State specific objectives, including any prespecified hypotheses	4 [85-90]	The Reversal of Type 2 diabetes …
**Methods**				
Study design	4	Present key elements of study design early in the paper	4 [104-111]	As monogenic ….
Setting	5	Describe the setting, locations, and relevant dates, including periods of recruitment, exposure, follow-up, and data collection	4 [93-103]	Participants with T2D of <6 years …were recruited by….
Participants	6	(*a*) *Cohort study*—Give the eligibility criteria, and the sources and methods of selection of participants. Describe methods of follow-up<br>*Case-control study*—Give the eligibility criteria, and the sources and methods of case ascertainment and control selection. Give the rationale for the choice of cases and controls<br>*Cross-sectional study*—Give the eligibility criteria, and the sources and methods of selection of participants	4 [109-111] &<br>4 [118-119]	The cohort of ….<br>were supervised by one-to-one contact ……
		(*b*)*Cohort study*—For matched studies, give matching criteria and number of exposed and unexposed<br>*Case-control study*—For matched studies, give matching criteria and the number of controls per case	4 [96-97]	normoglycemic control participants with no family….
Variables	7	Clearly define all outcomes, exposures, predictors, potential confounders, and effect modifiers. Give diagnostic criteria, if applicable	4[122-165]	Diary-based total dietary….
Data sources/ measurement	8*	For each variable of interest, give sources of data and details of methods of assessment (measurement). Describe comparability of assessment methods if there is more than one group	4[122-165]	Diary-based total dietary….
Bias	9	Describe any efforts to address potential sources of bias	4[104-108]	As monogenic and autoimmune.......
Study size	10	Explain how the study size was arrived at	6 [167-191]	Power calculation: The study was powered ……

Quantitative variables	11	Explain how quantitative variables were handled in the analyses. If applicable, describe which groupings were chosen and why	6 [180-182]	Analyses were conducted …..,.
Statistical methods	12	(*a*) Describe all statistical methods, including those used to control for confounding	6 [180-182]	Power calculation: ….
		(*b*) Describe any methods used to examine subgroups and interactions		
		(*c*) Explain how missing data were addressed	7 [190-112]	Missing data are indicated …….
		(*d*) *Cohort study*—If applicable, explain how loss to follow-up was addressed<br>*Case-control study*—If applicable, explain how matching of cases and controls was addressed<br>*Cross-sectional study*—If applicable, describe analytical methods taking account of sampling strategy	7 [190-112]	Missing data are indicated …….
		(*e*) Describe any sensitivity analyses		
**Results**				
Participants	13*	(a) Report numbers of individuals at each stage of study—eg numbers potentially eligible, examined for eligibility, confirmed eligible, included in the study, completing follow-up, and analysed	4 [109-110] & in Figures + Tables pages 18-24	The cohort of 20 …
		(b) Give reasons for non-participation at each stage	7 [5188-190]	Three participants were recruited…
		(c) Consider use of a flow diagram		
Descriptive data	14*	(a) Give characteristics of study participants (eg demographic, clinical, social) and information on exposures and potential confounders	7 [195-204] & [202-203] + Table 1	At baseline, there were 20 individuals ……. &<br>They were 58.0±10.5 years ….
		(b) Indicate number of participants with missing data for each variable of interest	17-23 in Figures & Tables	
		(c) *Cohort study*—Summarise follow-up time (eg, average and total amount)	4 [109-110]	The cohort of 20 people…
Outcome data	15*	*Cohort study*—Report numbers of outcome events or summary measures over time	4-9 [194-195 et seq]	Of 24 subjects studied …..
		Case-control study—Report numbers in each exposure category, or summary measures of exposure		
		Cross-sectional study—Report numbers of outcome events or summary measures		
Main results	16	(*a*) Give unadjusted estimates and, if applicable, confounder-adjusted estimates and their precision (eg, 95% confidence interval). Make clear which confounders were adjusted for and why they were included	4-9 [206-195 et seq]	In the T2D group mean body weight…..
		(*b*) Report category boundaries when continuous variables were categorized		
		(*c*) If relevant, consider translating estimates of relative risk into absolute risk for a meaningful time period		

Continued on next page

Other analyses	17	Report other analyses done—eg analyses of subgroups and interactions, and sensitivity analyses		
**Discussion**				
Key results	18	Summarise key results with reference to study objectives	14 [404-406 and 409-418]	The Personal Fat Threshold Hypothesis ….<br>Clinical Perspectives …
Limitations	19	Discuss limitations of the study, taking into account sources of potential bias or imprecision. Discuss both direction and magnitude of any potential bias	14 [289-303]	Limitations of the present…..
Interpretation	20	Give a cautious overall interpretation of results considering objectives, limitations, multiplicity of analyses, results from similar studies, and other relevant evidence	10 [199-224]	The ReTUNE study confirms ….
Generalisability	21	Discuss the generalisability (external validity) of the study results	13 [389-403]	Limitations ….
**Other information**				
Funding	22	Give the source of funding and the role of the funders for the present study and, if applicable, for the original study on which the present article is based	15 [439-441]	The study was funded ……

*Give information separately for cases and controls in case-control studies and, if applicable, for exposed and unexposed groups in cohort and cross-sectional studies.

Note: An Explanation and Elaboration article discusses each checklist item and gives methodological background and published examples of transparent reporting. The STROBE checklist is best used in conjunction with this article (freely available on the Web sites of PLoS Medicine at http://www.plosmedicine.org/, Annals of Internal Medicine at http://www.annals.org/, and Epidemiology at http://www.epidem.com/). Information on the STROBE Initiative is available at www.strobe-statement.org.

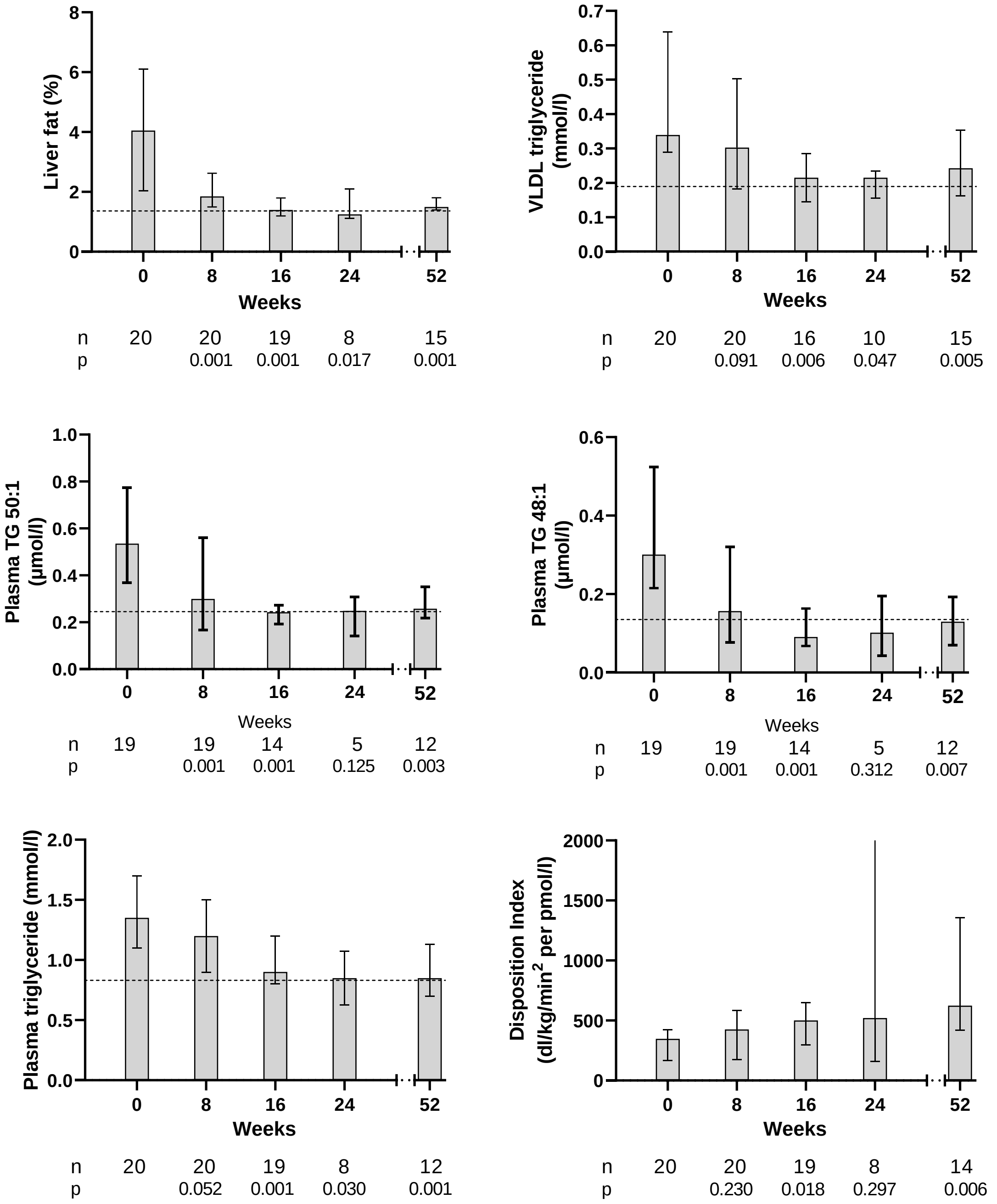

Figure S1

Time sequence of change in major pathophysiologic factors. Data are median (IQR) with number of participants at each data point and statistical significance compared with baseline. Correction for multiple testing did not cause loss of significant difference.

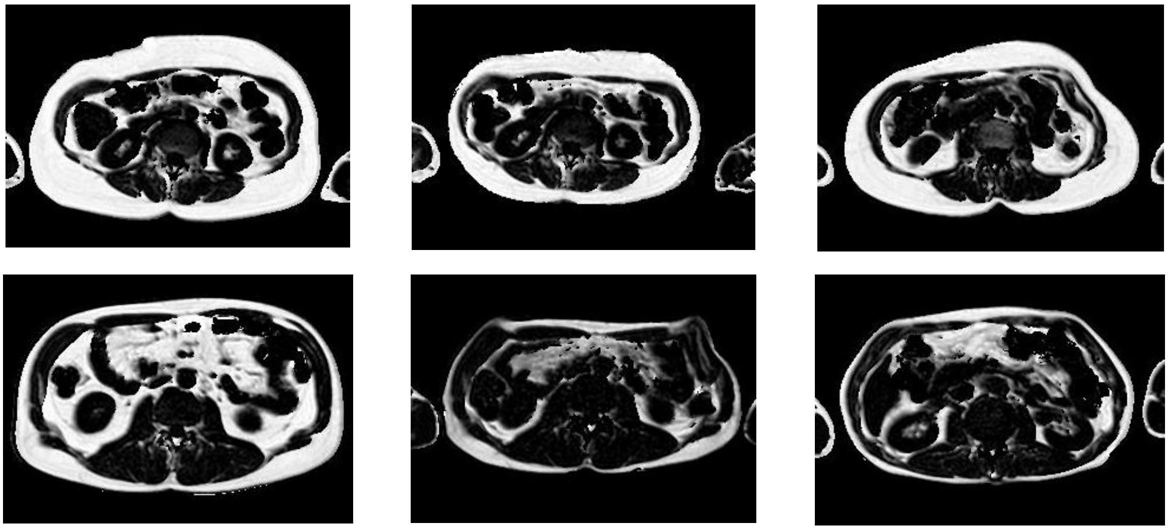

Figure S2:

Fat fraction maps of the abdomen at L2/3 level derived from Dixon MRI scans. The change in visceral fat in the T2D participants is demonstrated. Fat is shown in white, and the decrease in fat inside the visceral cavity induced by weight loss can be appreciated. Top row: (*left)* Female T2D participant at baseline (BMI 24.3) and (*centre)* 12 months (BMI 22.2); (*right)* Female control (BMI 22.2). Bottom row: (*left)* Male T2D participant at baseline (BMI 26.0) and (centre) 12 months (BMI 23.2);(*right*) Male control (BMI 21.2).

Table S1: Change in NEFA (mmol/l; mean±SEM) during the standard meal test (time in minutes from start of meal). For each time point of the meal test statistical significance is shown compared with the baseline (B0) meal test data.

**Time**	**-15**	**30**	**90**	**180**
**Baseline**	0.72±0.04	0.56±0.02	0.18±0.02	0.13±0.05
**8 weeks**	0.60±0.05<br>p=0.077	0.57±0.05<br>p=0.844	0.17±0.02<br>p=0.581	0.10±0.01<br>p=0.059
**16 weeks**	0.61±0.04<br>p=0.185	0.55±0.04<br>p=0.948	0.17±0.02<br>p=0.503	0.12±0.02<br>p=0.579
**24 weeks**	0.82±0.13<br>p=0.732	0.55±0.07<br>p=0.999	0.14±0.02<br>p=0.469	0.22±0.15<br>p=0.448
**52 weeks**	0.57±0.04<br>p=0.155	0.48±0.05<br>p=0.449	0.16±0.03<br>p=0.020	0.10±0.01<br>p=0.446

Table S2: Baseline clinical characteristics of those who achieved diabetes remission compared with those who did not (mean±SD). No glucose lowering agents apart from metformin and gliclazide were being taken at baseline.

	**Remission group (n=14)**	**Non-remission group (n=6)**	**p**
Age (years)	59.0±7.0	59.0±7.5	0.990
Sex (F/M)	8/6	5/1	0.260
Weight (kg)	74.5 ±13.8	65.6±6.0	0.152
BMI (kg/m^2)	25.1±1.7	24.1±1.7	0.275
Ethnicity:			
White European	11	5	0.861
Asian	2	1	
Middle Eastern	1	0	
Known duration of diabetes (years)	2.5±2.1	3.4±1.4	0.322
1st degree family history (n)	8	3	0.861
HbA_{1c} (mmol/mol)	54±7	54.0±4	0.981
HOMA_IR	2.02(1.51-3.24)	1.74(1.06-4.35)	0.621
Glucose-lowering agents (n)	Metformin 5<br>Gliclazide 1	Metformin 5<br>Gliclazide 1	0.100
Systolic BP (mmHg)	133±4	123±6	0.137
Diastolic BP (mmHg)	77±3	67±3	0.035
One antihypertensive<br>Two antihypertensives	3<br>1	0<br>0	0.143

Table S3: Composition of average daily food intake over 3 days (2 weekdays and 1 weekend day) during final 2 weeks of each stage as self-reported using Intake24. Compliance with completing the diaries decreased during the study. Data are mean and SEM. For each timepoint statistical significance is shown compared with baseline.

	**Baseline** (n=16)	**8 weeks** (n=14)	**16 weeks** (n=8)	**24 weeks** (n=4)	**52 weeks** (n=8)
Energy (kcal/day)	1463.5±118.2	1197 ± 110 p=0.0116	1065±172 p=0.0185	684±246 p=0.1344	1108±118 p=0.0192
Protein (g/day)	61.8±4.3	59.8±5.9 p=0.4734	55.6±7.8 p=0.1570	60.8±3.0 p=0.7001	58.4±5.1 p=0.2622
Carbohydrate (g/day)	159.6±3.0	112.2±5.3 p=0.0001	104.8±5.5 p=0.0038	95.3±28.0 p=0.0886	126.0±6.8 p=0.0273
Fat (g/day)	60.6±6.3	54.7±7.0 p=0.2575	44.7±10.7 p=0.1293	40.3±6.4 p=0.2206	41.0±4.5 p=0.0524
Saturated Fat (g/day)	21.7±2.9	17.3±2.6 p=0.1829	13.0±2.7 p=0.0537	11.2±2.0 p=0.0728	10.9±2.1 p=0.0123
Cis Mono-unsaturated (g/day)	21.5±2.7	21.1±3.2 p=0.6371	18.0±5.2 p=0.4004	15.1±2.4 p=0.4440	16.0±1.8 p=0.1601
Cis Poly- unsaturated (g/day)	9.6±1.2	10.6±1.2 p=0.9503	9.5±2.5 p=0.7295	9.7±2.4 p=0.8787	9.9±1.1 p=0.7011
Fibre (Englyst) (g/day)	12.8±1.4	13.8±2.0 p=0.8864	13.5±1.9 p=0.3382	11.7±2.2 p=0.8546	14.1±4.3 p=0.8815
Trans FA (g/day)	0.8±0.1	0.7±0.1 p=0.3258	0.6±0.1 p=0.1802	0.6±0.1 p=0.1142	0.3±0.1 p=0.0225
Fruit portions (n)	1.0±0.3	2.0±0.5 p=0.1323	1.1±0.6 p=0.5529	0.6±0.5 p=0.0309	1.9±0.7 p=0.2105
Vegetable portions (80g) (n)	1.7±0.3	2.6±0.5 p=0.0066	2.7±0.7 p=0.1579	2.8±0.3 p=0.0632	2.5±0.8 p=0.3148
